# Riboflavin^☆^

**DOI:** 10.1016/j.advnut.2026.100649

**Published:** 2026-05-12

**Authors:** John Thomas Pinto, Lora A Sporny

**Affiliations:** 1 Department of Cell and Molecular Physiology, New York Medical College, Valhalla, NY, United States; 2 Department of Health Studies & Applied Educational Psychology Teachers College, Columbia University, New York, NY, United States

**Keywords:** riboflavin metabolism, flavoproteins, mitochondrial function, riboflavin deficiency, colorectal cancer, immune modulation

Riboflavin (vitamin B-2), first identified in the late 19th century as the yellow milk pigment lactochrome, was structurally defined by the 1930s as an isoalloxazine ring attached to ribitol, establishing its role as the precursor of riboflavin-5′-phosphate (FMN) and FAD. Flavoproteins predominantly catalyze redox reactions, enabling electron transport, lipid and xenobiotic metabolism, signaling, and protein folding. Recent research highlights essential contributions of riboflavin to mitochondrial energy production, antioxidant defense, and metabolic regulation through FMN- and FAD-dependent pathways. Advances in the biology of riboflavin transporters (SLC52A1–A3) also reveal how impaired riboflavin uptake or flavoprotein assembly promotes metabolic and neurological disease. Together, these developments establish riboflavin as a central node connecting coenzyme biogenesis, cellular redox control, and systems-level metabolism.

The ATP-binding cassette G2 transporter (ABCG2), originally identified as the breast cancer resistance protein, is a multidrug transporter that exports riboflavin into breast milk and other extracellular fluids such as bile, cerebrospinal fluid, and semen. Its expression rises sharply during lactation and declines after weaning, aligning with its role in delivering flavins to the infant; mature breast milk contains ∼650 μg/L total flavins, mainly FAD (54%) and riboflavin (39%). Emerging evidence shows that ABCG2 also governs the secretion and tissue distribution of catabolic riboflavin derivatives (e.g., lumichrome), underscoring its broader function in regulating vitamin B_2_ bioavailability and its importance as a biomarker substrate in transporter research. Accordingly, ABCG2 activity influences both nutrient transfer to the neonate and systemic handling of riboflavin and its metabolites.

Riboflavin is converted to its active coenzymes through phosphorylation by flavokinase (ATP:riboflavin 5-phosphotransferase) to produce FMN, followed by an AMP-dependent reaction by FAD synthetase (ATP:FMN adenylyl transferase) to form FAD. Thyroid hormones enhance expression of these enzymes across mammalian tissues. Recent structural work identifies human FAD synthetase as a bifunctional, multidomain enzyme with distinct catalytic sites for FAD synthesis and hydrolysis, offering new insight into coenzyme homeostasis. These regulatory layers ensure coordinated FMN/FAD production to match cellular demand and preserve redox balance.

Riboflavin is vital for 1- and 2-electron transfer reactions within the mitochondrial electron transport chain. Complexes I and II depend on flavoprotein dehydrogenases and electron-transferring flavoproteins, whereas complexes III and IV rely on ubiquinone and cytochromes. Complex I defects are major contributors to mitochondrial disorders associated with neurodegeneration. Recent work reinforces the importance of FMN and FAD in sustaining mitochondrial energy production, redox homeostasis, and neuronal function, linking disruptions in riboflavin metabolism or transport to a widening spectrum of neurological conditions, including migraine, multiple acyl-CoA dehydrogenase deficiency (MADD), riboflavin transporter deficiencies (RTDs), Parkinson’s and Alzheimer’s disease, multiple sclerosis, and acute brain injury [1,2]. This continuum highlights how perturbed flavin biology can propagate from organelle dysfunction to clinical neurodegeneration.

## Deficiencies

Disorders that impair intestinal absorption, including malabsorptive conditions or bowel resection, can produce clinically significant riboflavin deficiency. Persistent deficiency also characterizes Brown Vialetto-Van Laere (BVVL) and Fazio-Londe syndromes, now unified as RTD caused by pathogenic variants in SLC52A1, SLC52A2, or SLC52A3. BVVL typically presents with sensorineural hearing loss, sensory ataxia, and progressive bulbar and respiratory dysfunction, whereas Fazio-Londe shares similar neurological features but lacks hearing loss. Early high-dose riboflavin therapy can stabilize or reverse disease progression and is often lifesaving, underscoring the importance of prompt recognition and intervention. Genotype-guided dosing strategies are increasingly used in RTD management.

Because flavoproteins support numerous pathways, riboflavin deficiency most severely disrupts lipid metabolism. FMN and FAD are required for transferase, dehydrogenase, oxidoreductase, monooxygenase, and oxidase activities that mediate fatty acid desaturation; phospholipid and ether lipid synthesis; and the production of sphingosine, cholesterol, and steroid hormones. Even marginal riboflavin deficiency can produce cutaneous manifestations resembling those of essential fatty acid deficiency and can impair hepatic mitochondrial β-oxidation. Recent studies show that low riboflavin status, particularly in the context of high-fat intake, worsens hepatic fat accumulation through altered Peroxisome Proliferator-Activated Receptor gamma (PPARγ) activity. Progressive deficiency further compromises FAD-dependent enzymes, exacerbating oxidative stress and endoplasmic reticulum (ER) stress, impairing mitochondrial function, and promoting lipotoxicity, defined as cellular injury caused by the accumulation of toxic lipid intermediates. Collectively, these disruptions amplify metabolic vulnerability and contribute to systemic dysfunction. These mechanisms integrate to explain the multisystem phenotype of riboflavin deficiency.

Riboflavin deficiency compromises antioxidant defenses by impairing the FAD‑dependent enzyme glutathione reductase, which is responsible for maintaining the intracellular redox balance. Under normal conditions, this balance keeps reduced glutathione (GSH) at much higher levels than oxidized glutathione (GSSG), typically in a ratio of ∼30:1 to 100:1 in healthy tissues. Oxidized glutathione is generated when two GSH molecules are converted into a disulfide bond during the detoxification of peroxides. As GSH becomes limited under oxidative stress, its availability as a cosubstrate for glutathione peroxidase and glutathione S*-*transferase is increasingly restricted, diminishing lipid peroxide detoxification and protein and xenobiotic *S-*glutathionylation. Consequently, inadequate riboflavin disrupts lipid and energy metabolism, redox control, and xenobiotic detoxification. Recent findings indicate that deficiency also promotes mitochondrial dysfunction, ER stress, and disordered lipid metabolism, collectively heightening oxidative injury and accelerating disease progression [3]. Thus, redox, lipid, and detoxification defects converge when riboflavin is insufficient.

Outside rare genetic or malabsorptive states, isolated riboflavin deficiency remains uncommon. Classic findings such as glossitis, angular stomatitis, cheilosis, and dermatitis are not pathognomonic and may occur in other nutrient deficiencies; riboflavin deficiency usually co-occurs with broader micronutrient inadequacy. Although overt deficiency is rare, emerging evidence suggests that suboptimal riboflavin status may be more widespread than previously recognized. Light-based medical treatments can further lower status in vulnerable groups (e.g., neonates), reinforcing the need for contextual assessment [4]. Beyond primary deficiency and impaired absorption, riboflavin status may be reduced by endocrine disorders such as aldosterone or thyroid hormone insufficiency, as well as medications including tricyclic antidepressants and tetracycline antibiotics. Excess alcohol intake also decreases riboflavin utilization. Clinical observations indicate that light-based medical treatments and metabolic disturbances affecting folate or homocysteine pathways can heighten physiological demand or accelerate loss, increasing susceptibility to insufficient riboflavin levels. Thus, multiple physiological, pharmacologic, and environmental factors can converge to compromise riboflavin homeostasis. A careful medication, diet, and exposure history helps identify these contributors.

## Diet Recommendations

NHANES 2021 to 2023 data indicate that inadequate riboflavin intake is uncommon in the United States, largely due to long-standing fortification of flour, cereals, breads, pasta, and rice. Across the life course, recommended riboflavin intakes are modest. Infant adequate intake (AI) values range from 0.3 to 0.4 mg/d, reflecting typical milk intake during early development. Childhood recommended dietary allowances (RDAs) increase progressively from 0.5 to 0.6 mg/d for ages 1 to 8 y to 0.9 mg/d for ages 9 to 13 y, whereas adolescent requirements range from 1.0 to 1.3 mg/d depending on sex. For adults aged 19 to 70+ y, RDAs are set at 1.3 mg/d for men and 1.1 mg/d for women, with increased requirements during pregnancy (1.4 mg/d) and lactation (1.6 mg/d). These benchmarks are informed by deficiency indicators observed below ∼0.6 mg/d and by erythrocyte glutathione reductase activation coefficients indicating functional adequacy ∼1.0 mg/d, thereby linking intake recommendations to established biochemical markers across life stages. Consistent with these standards, children and adolescents aged 2 to 19 y generally meet riboflavin requirements, with mean intakes ranging from 1.35 mg/d in non-Hispanic Black youth to 1.75 mg/d in non-Hispanic White youth, whereas adults typically consume ∼1.51 to 2.07 mg/d; overall intake among individuals aged ≥2 y remains within the RDA range of 1.47 to 2.01 mg/d [5].

Earlier NHANES assessments (1988–1994) similarly identified riboflavin inadequacy as uncommon, reporting no deficiencies among children aged 2 to 8 y, modest shortfalls in ∼5% of adolescent girls, and prevalence estimates of roughly 2% among adults. Subsequent survey cycles, including NHANES 2005 to 2016 and 2017 to 2018, have consistently supported these observations, indicating that most United States residents continue to achieve recommended intake levels across age groups. Higher riboflavin intake has also been associated with favorable cardiovascular and coronary heart disease outcomes, underscoring the relevance of sustaining AI throughout adulthood. Nonetheless, consideration of riboflavin status may remain warranted for select subgroups based on dietary patterns, health status, or clinical context.

## Food Sources

Flavins occur naturally and are widely distributed across plant and animal foods. Organ meats, poultry, fish, eggs, and dairy products are particularly rich in the coenzymic forms, whereas cereals and grains are widely fortified with free riboflavin in the United States and many other countries. In many low-income settings, legumes, tofu, whole grains, and whole grain breads and cereals serve as primary contributors to overall flavin intake, with green vegetables (e.g., broccoli, Brussels sprouts, spinach, beet, collard, and turnip greens, and asparagus) providing moderate amounts. Because the flavins are water soluble and heat sensitive, preparation methods such as steaming, roasting, stir frying, or microwaving are preferred over boiling, and overcooking should be avoided. Fortification markedly increases the contribution of grains to total flavin intake, making fortified cereals, dairy products, and organ meats among the most reliable dietary sources.

Protein-dense foods provide riboflavin alongside other B vitamins, reinforcing their role within integrated micronutrient networks. Because flavin-dependent enzymes support folate, vitamin B6, niacin metabolism, vitamin K recycling, and vitamin D activation, riboflavin deficiency often co-occurs with broader micronutrient insufficiency. Even modest depletion can impair multiple biochemical pathways related to coagulation, bone health, and one-carbon metabolism. This interdependence underscores the importance of dietary patterns that supply riboflavin together with complementary nutrients.

Riboflavin is highly light sensitive, and exposure to UV or visible light substantially accelerates its degradation in food systems. This photosensitivity is a primary determinant of losses during food processing, storage, and preparation. Sun drying fruits and vegetables, for example, produces marked riboflavin loss through UV-driven photo oxidation, with degradation proportional to cumulative light dose. Similarly, dairy products stored in clear containers exhibit significantly higher riboflavin losses than those packaged in opaque materials, as fluorescent and UV radiation readily penetrate transparent packaging. Thus, packaging design plays a central role in preserving riboflavin content, particularly in milk and other light-exposed foods.

In practice, shielding foods from light during processing, storage, and display remains one of the most effective strategies for riboflavin preservation. Alkaline conditions, such as those introduced by baking soda during vegetable preparation, further accelerate photodegradation, whereas prolonged exposure to water or light during blanching, milling, fermenting, or extrusion increases losses. By contrast, riboflavin generally tolerates thermal processing, microwaving, and infrared heating, which distribute heat more evenly and limit surface exposure. However, extended heating, high temperatures, or alterations in pH can destabilize flavin protein complexes, indirectly increasing vulnerability to light.

When riboflavin is present in foods as part of protein-bound coenzymes (FMN and FAD), interactions between aromatic amino acids and the isoalloxazine ring help shield it from light-induced degradation. This protective effect is functionally important in protein-dense foods, where bound flavins exhibit greater stability than free riboflavin. Consequently, minimizing water contact, reducing alkaline additives, favoring gentle heat, and limiting light exposure collectively optimize riboflavin retention during food preparation. Overall, riboflavin is stable under most common cooking practices but remains distinctly vulnerable to light, alkaline environments, and prolonged heating [6]. Thus, packaging, processing method, pH, and light exposure interact synergistically to determine final riboflavin content in foods.

## Clinical Uses

In contrast to food systems, light–riboflavin interactions *in vivo* have distinct physiological and clinical implications. Exposure to UV or visible light, including prolonged therapeutic phototherapy, can accelerate riboflavin degradation within the body and contribute to depletion, particularly in vulnerable populations such as infants or individuals with marginal nutritional status. Under these circumstances, monitoring riboflavin status during extended light-based treatments may be advisable to reduce the risk of subclinical deficiency.

At pharmacologic doses exceeding ∼100 mg, riboflavin exhibits pronounced photo reactivity, as the isoalloxazine ring absorbs UV (≈380 nm) or blue visible light (≈450 nm) and generates reactive oxygen species. This photochemical activity leads to the formation of peroxides, secondary oxidants, and a characteristic tryptophan–riboflavin photo adduct with documented cytotoxic and hepatotoxic properties. These reactive intermediates preferentially damage light-exposed tissues, particularly the retina and lens, where elevated free riboflavin levels combined with intense illumination amplify oxidative stress and tissue injury. Although riboflavin is considered safe at nutritional intake levels, the combination of very high supplemental doses and sustained or intense light exposure warrants caution [7,8]. In summary, *in vivo* riboflavin photo reactivity is primarily a concern under conditions of pharmacologic dosing or prolonged therapeutic light exposure, where accelerated degradation and oxidative injury may compromise tissue integrity or micronutrient status.

Beyond its established photoreactive uses, such as pathogen inactivation and corneal cross-linking in keratoconus, recent work highlights a rapidly expanding therapeutic role for riboflavin across multiple disease states. High-dose riboflavin shows strong efficacy in neurological disorders including RTD, MADD, and migraine, with demonstrated improvements in mitochondrial function, redox balance, and neuronal viability. In pediatric RTD, >80% of patients exhibit recovery in motor, sensory, bulbar, and respiratory function, making riboflavin a genuinely lifesaving therapy. These outcomes illustrate how targeted cofactor repletion can restore impaired flavoprotein pathways.

Furthermore, riboflavin demonstrates genotype-specific benefit in blood pressure regulation. Individuals with the methylenetetrahydrofolate reductase (MTHFR) 677TT variant experience significant reductions in systolic and diastolic blood pressure with riboflavin supplementation, representing a low-cost, targeted approach to cardiovascular risk reduction [9]. This genotype-responsive effect reflects an emerging precision-nutrition role for riboflavin that is biologically distinct from its photosensitivity yet reinforces the importance of maintaining adequate, stable riboflavin status.

Riboflavin is also emerging as a targeted treatment for adult-onset metabolic disorders driven by flavoprotein gene variants, where many individuals experience clinically significant symptomatic improvement, underscoring its value in precision-nutrition and metabolic-management strategies. Additional evidence shows that riboflavin enhances mitochondrial bioenergetics and antioxidant defenses in neurodegenerative diseases, such as Parkinson’s and Alzheimer’s disease, suggesting therapeutic potential beyond classical deficiency [10,11]. Future trials will clarify optimal dosing, duration, and patient selection.

## Toxicity

Orally consumed riboflavin, whether obtained from food or standard supplemental doses, rarely produces adverse effects or reaches toxic concentrations, as free riboflavin circulates only briefly before being efficiently excreted in urine or exported into extracellular fluids via ABCG2. This rapid clearance, combined with limited intestinal absorptive capacity at higher intakes, constrains systemic accumulation and contributes to its strong overall safety profile. Accordingly, no tolerable upper intake level has been established for typical dietary use.

Although routine supplementation remains well tolerated, extremely high or repeated pharmacologic intakes may increase the pool of unbound riboflavin available for photochemical reactions under certain environmental conditions. In such settings, excess free riboflavin can enhance susceptibility to oxidative processes, reinforcing the general recommendation to avoid unnecessarily large supplemental doses when no clinical indication exists. Beyond optical effects described in the light-sensitivity section, no additional systemic toxic syndromes have been identified. Minor and transient changes, namely, bright yellow discoloration of urine, reflect renal excretion of unmetabolized riboflavin and are not considered harmful. Because ABCG2 facilitates export of surplus riboflavin into extracellular fluids, high oral intakes do not typically overwhelm metabolic pathways or result in tissue accumulation. Thus, meaningful safety concerns arise only under uncommon conditions involving unusually high levels of exposure.

Riboflavin is not commonly included in mainstream self-tanning products, although some tanning accelerators incorporate it with acetyl-tyrosine or other melanogenic cofactors to enhance UV-induced pigment formation. Such cosmetic uses rely on its photoreactive properties but have not been associated with systemic toxicity due to minimal dermal penetration and limited total exposure. Available evidence does not indicate meaningful risk from these topical applications.

## Recent Research

Recent investigations position riboflavin as a multifunctional immunonutrient with clinical relevance across health domains. In colorectal cancer, tumor cells upregulate SLC52A2 to deplete local riboflavin, thereby starving CD8^+^ T cells of a cofactor required for cytotoxic function. Using organoids, metabolomics, *in vitro* assays, and murine models, researchers show that riboflavin supplementation does not fuel tumor growth; instead, it replenishes intratumoral riboflavin, rescues CD8^+^ effector capacity and viability, and suppresses tumor progression. These findings identify riboflavin deprivation as an immune-evasion mechanism and support riboflavin as a translatable, immunonutritional strategy to strengthen antitumor immunity in colorectal cancer [12]. This work reframes riboflavin as an immune-supportive cofactor in the tumor microenvironment.

Beyond the use of riboflavin in maintenance of host nutrition, emerging research is examining human microbiota (commensal bacteria and fungi) that generate *de novo* riboflavin-derived metabolites capable of activating mucosa-associated invariant T (MAIT) cell development and immune function. MAIT cells colonize mucosal and barrier tissues such as the gut, lung, and oral cavity. Several intermediates in the riboflavin biosynthetic pathway, notably 5-(2-oxopropylideneamino)-6-D-ribitylaminouracil, act as potent major histocompatibility complex class I-related protein 1-binding ligands capable of activating MAIT cells. These riboflavin-derived metabolites can be harnessed to selectively expand and reprogram MAIT cells toward a proinflammatory, antiviral MAIT1 phenotype, thereby enhancing both innate and adaptive immune responses, including CD8^+^ T-cell activation. Because viruses cannot generate these metabolites themselves, their microbial origin provides a unique immunomodulatory lever that can be exploited to strengthen vaccine efficacy and antiviral defense. This emerging line of research highlights riboflavin-pathway intermediates as promising immunological tools with potential applications in next-generation vaccine adjuvant design [13]. Collectively, these findings connect diet–microbe interactions with host antiviral immunity through riboflavin-linked pathways.

## Author contributions

The authors’ responsibilities were as follows—JTPperformed the literature review, wrote and designed the article; LAS was involved in editing and providing nutritional expertise regarding diet recommendations and food sources; and both authors have read and approved the final manuscript.

## Declaration of generative AI and AI-assisted technologies in the writing process

The authors declare that no generative AI or AI-assisted technologies were used in the writing of this manuscript.

## Funding

The authors reported no funding was received for this study.

## Conflict of interest

The authors report no conflicts of interest.
